# MicroRNAs in Cervical Cancer: Evidences for a miRNA Profile Deregulated by HPV and Its Impact on Radio-Resistance

**DOI:** 10.3390/molecules19056263

**Published:** 2014-05-16

**Authors:** Abraham Pedroza-Torres, Eduardo López-Urrutia, Verónica García-Castillo, Nadia Jacobo-Herrera, Luis A. Herrera, Oscar Peralta-Zaragoza, César López-Camarillo, David Cantú De Leon, Jorge Fernández-Retana, Jorge F. Cerna-Cortés, Carlos Pérez-Plasencia

**Affiliations:** 1 Instituto Nacional de Cancerología, Laboratorio de Genómica, Mexico DF 14080, Mexico; E-Mails: abraneet@gmail.com (A.P.-T.); e_urrutia@me.com (E.L.-U.); ritepjorge.mivado@gmail.com (J.F.-R.); 2 Universidad Nacional Autónoma de México UNAM, FES-Iztacala, UBIMED, Tlalnepantla, Estado de México 54090, Mexico; E-Mail: veronica_garcia_367@hotmail.com; 3 INCMNSZ, Unidad de Bioquímica, Mexico DF 14080, Mexico; E-Mail: nadia.jacobo@gmail.com; 4 Unidad de Investigación Biomédica en Cáncer, Instituto Nacional de Cancerología (INCan)-Instituto de Investigaciones Biomédicas, UNAM, Mexico DF 14080, Mexico; E-Mails: herreram@biomedicas.unam.mx (L.A.H.); dcantude@gmail.com (D.C.D.L.); 5 Instituto Nacional de Salud Pública, INSP. Centro de Investigación en Enfermedades Infecciosas, CISEI, Cuernavaca 62100, Mexico; E-Mail: operalta@correo.insp.mx; 6 UACM, Posgrado en Ciencias Genómicas, Mexico DF 06720, Mexico; E-Mail: cesar.lopez@uacm.edu.mx; 7 Instituto Politécnico Nacional, Escuela Nacional de Ciencias Biologicas, Departamento de Microbiología, México DF 07738, Mexico; E-Mail: jcerna@hotmail.com; 8 Instituto Nacional de Cancerología, Unidad de Genómica y Secuenciación Masiva (UGESEM-INCan), Mexico DF 14080, Mexico

**Keywords:** miRNAs, mirnome, cervical cancer, HPV, radio-resistance

## Abstract

Cervical carcinoma (CC) is one of the most common cancers and a leading cause of mortality in women worldwide. Epidemiologic and experimental data have clearly demonstrated a causal role of high-risk Human Papillomavirus (HR-HPV) types in CC initiation and progression, affecting the cellular processes by targeting and inactivating p53 and pRB host proteins. HR-HPV E5, E6 and E7 oncoproteins have the ability to deregulate several cellular processes, mostly apoptosis, cell cycle control, migration, immune evasion, and induction of genetic instability, which promote the accumulation of mutations and aneuploidy. In this scenario, genomic profiles have shown that aberrant expression of cellular oncogenic and tumor suppressive miRNAs have an important role in CC carcinogenesis. It has been stated that HPV infection and E6/E7 expression are essential but not sufficient to lead to CC development; hence other genetic and epigenetic factors have to be involved in this complex disease. Recent evidence suggests an important level of interaction among E6/E7 viral proteins and cellular miRNA, and other noncoding RNAs. The aim of the current review is to analyze recent data that mainly describe the interaction between HR-HPV established infections and specific cellular miRNAs; moreover, to understand how those interactions could affect radio-therapeutic response in tumor cells.

## 1. Introduction

**Figure 1:**
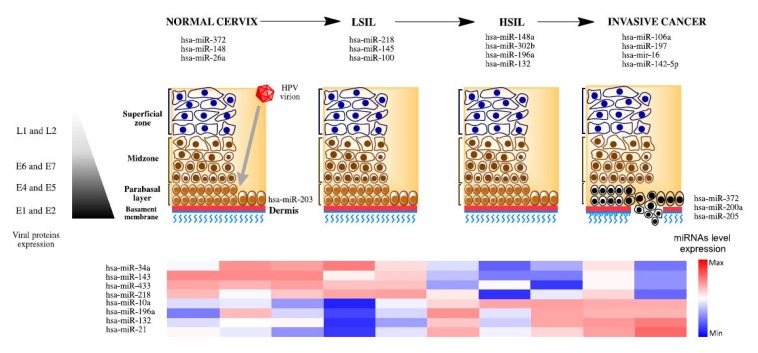
Expression of miRNAs through the cervical cancer progression. Human papillomavirus infects epithelial basal cells through mechanical microabrassions or by infecting directly the transformation zone, an abrupt transition from columnar to squamous epithelium. Infected parabasal cells actively express the early genes E1, E2, E4, and E5, E6 and E7 are expressed in limited quantities due to transcriptional modulation exerted by E2, which allows cells to have a higher cell-cycle progression. Infected basal cells migrate to the lumen as they differentiate expressing the late capsid genes L1 and L2. Several miRNAs have been associated with different clinical stages, including normal tissue, premalignant lesions (low and high grade squamous intraepithelial lesion, LSIL and HSIL, respectively) and invasive cancer represented at the bottom of the figure.

Cervical cancer (CC) is the third most common cancer in women around the world, representing 9.8% of all female cancers; with an estimated 530,000 new cases in 2008. More than 85% of the CC global burden occurs in developing countries, where it accounts for 13% of all female cancers. High-risk regions are Eastern and Western Africa (greater than 30 cases per 100,000 inhabitants), Southern Africa (26.8 per 100,000), South-Central Asia (24.6 per 100,000), South America and Middle Africa (23.9 and 23.0 per 100,000, respectively). Cervical cancer remains the most common cancer in women only in Eastern Africa, South-Central Asia and Melanesia. During 2008, CC was responsible for 275,000 death, about 88% of which occurred in developing countries distributed in Africa with 53,000 cases, 31,700 in Latin America and the Caribbean, and 159,800 in Asia [1]. Clearly, clinical, epidemiological and molecular data have shown that persistent human papillomavirus (HPV) infection is the main etiological agent in CC development [2]. Anogenital cancers, including cervical cancer (CC), are associated with high risk HPV types (mainly 16 and 18) [3]. During the cervical carcinogenesis process E5, E6 and E7 from high-risk HPV (HR-HPV) play a major role by inactivation of cellular mechanisms involved in the control of cell cycle. However, it has been postulated that HPV is a necessary—but not sufficient—cause for developing cervical carcinoma; thus, other types of factors such as cellular, immunological, genetic, epigenetic, or environmental, can affect the final outcome of the disease [3,4,5] (Figure 1).

Cervical cancer patients are treated by surgery and chemo- and radiotherapy approaches, although radical hysterectomy is employed in early stages. Radiotherapy in combination with cisplatinum is the standard treatment for invasive cervical cancer [6,7]. Therefore, radio-chemotherapy has become the major non-surgical treatment in locally advanced cervical cancer (LACC) patients. Nonetheless, radio- and chemoresistance are the major obstacles to efficiently treat cervical cancer and aggressive tumors in general. LACC patients treated with radiotherapy have in general a 50% chance of recurrence or persistent disease, which could be explained by the presence of radio-resistant cells inside the tumor mass. Recently, a meta-analysis showed that CC patients receiving radiotherapy alone or in combination with different chemotherapeutic agents, had at five years an overall survival rate ranging from 40%–70% [8]. Several global meta-analyses, including patients treated in developed and developing countries, have shown similar results [9,10]. Here, we explore the hypothesis that HR-HPV can affect the expression levels of cellular miRNAs that could be involved, not only in the establishment of malignant phenotype, but also in the resistance to radio- and chemotherapy in CC patients.

## 2. MiRNA Biogenesis

**Figure 2:**
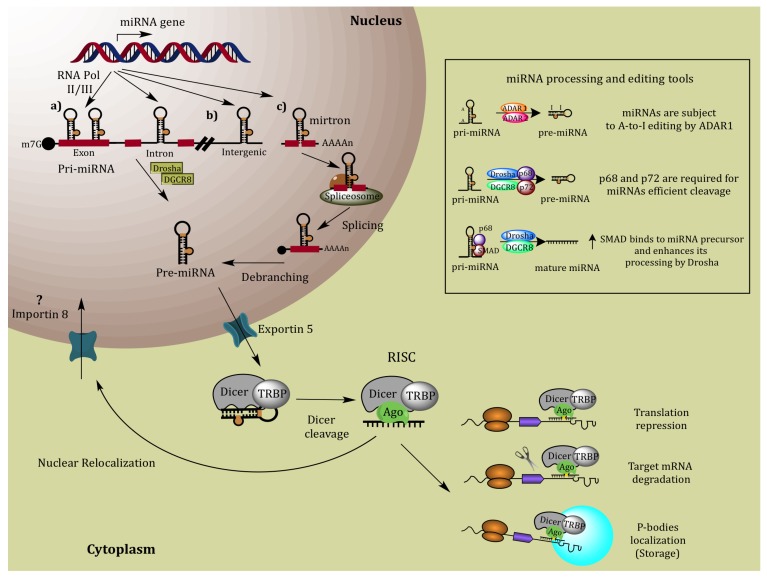
Canonical and non-canonical miRNA biogenesis pathways. RNA pol II transcribes a primary transcript called pri-miRNA which contains a 7-methylguanosine cap and poly(A) tail. Subsequently, this structure is degraded into the nucleus by an RNase type III known as Drosha; producing 70 nt long pre-miRNAs, which are exported to the cytoplasm by exportin-5. Once in the cytoplasm, pre-miRNAs are cleaved by RNase III endonuclease known as Dicer and associated proteins TRBP and PACT, resulting in a double stranded molecule called miRNA duplex. Then, the level of complementarity between miRNAs with their target mRNA could affect the following mechanism: if presented a total complementarity, mRNA degradation is carried out; on the contrary, if complementarity is partial, the inhibition of mRNA translation occurs.

MicroRNAs or miRNAs are single-stranded RNAs constituted by around 21-23 nucleotides. They are considered small non-coding RNAs, which can inhibit gene expression through transcriptional repression and degradation of canonical mRNA (for instance, protein-coding messenger RNAs); miRNAs are present in animals, plants and unicellular eukaryotes [11,12]. miRNA biosynthesis involves the generation of hairpin-shaped long transcripts by RNA polymerase II (RNA pol II); miRNA genes are initially transcribed to yield a primary, long transcript that undergoes successive processing in both the nucleus and cytoplasm by type III ribonucleases known as Drosha (in nuclei) and Dicer (in cytoplasm). Most miRNAs genes are located in intergenic non-coding regions, but they could also be found within exonic or intronic regions, in either sense or antisense orientation, and furthermore, could be independently transcribed from their own promoters [13,14] (Figure 2a–c). miRNAs located within intron regions of protein-coding or non-coding genes (pseudo-genes) have been denominated “mirtrons” [15]. MiRNAs are grouped in families depending on their sequence similarity and function, and can be localized as single units or arranged in clusters in the genome. It has been estimated that a single miRNA can negatively regulate hundreds, or even thousands, of target genes [16]. However, function and cellular targets of most of miRNAs remain to be determined.

Canonical miRNA biogenesis pathway is initiated by RNA pol II; producing a long primary transcript called primary miRNA (pri-miRNA). Clustered miRNAs are transcribed from a single transcription unit as a polycistronic pri-miRNA. Pri-miRNAs resemble canonical mRNA, due to they have both 5'-cap structure (7MGpppG) as well as 3'-end polyadenylated tails (Poly-A) [17]. Then, synthesized pri-miRNAs are transported to the cytoplasm, where they are processed by another RNAse III enzyme known as DICER (dicing process); that in coordination with dsRNA-binding protein TRBP2, cuts out the loop and generates a double stranded RNA formed by a guide (miRNA) and a transient strand (miRNA*) which is degraded by the Argonaut protein 2 (AGO2), required for miRNA-induced silencing. Dicer, AGO proteins and mature miRNA, form an RNA-induced silencing complex (RISC) leading to the creation of a miRNA-induced silencing complex (miRISC), which acts as a guide to deliver the complex to target mRNA [18,19]. Then, the mature microRNA hybridizes to nearly-complementary sites in the 3' un-translated region (3'-UTR) of the target mRNA.

Mature miRNAs negatively regulate gene expression by complementarity between a given miRNA and its target gene; however the involved mechanism depends upon the level of complementarity between the miRNA and its target mRNA [20]. Gene silencing can be achieved by means of mRNA degradation or by inhibiting the mRNA from being translated. Translational repression of transcripts is driven when miRNA binds to target with imperfect complementarity, as seen in the majority of cases. This imperfect miRNA: mRNA interaction means that a single miRNA can potentially target tens to hundreds of mRNAs. When a miRNA binds to its mRNA targets with a high complementarity, the degradation of the messenger is induced with the participation of the RISC complex [21] (Figure 2).

Both, translation repression and degradation of mRNA targets by miRNAs, occurs in specific cytoplasmic foci known as mRNA processing bodies (P-bodies), which are enriched in ribonucleoproteins (RNPs), mRNA decay factors, and miRNAs [22]. P-bodies thus represent the cytoplasmic mRNA processing centers, where non-translating transcripts are sorted and silenced, or degraded. P-bodies observed in yeasts, insects, nematodes and mammalian cells have critical roles in mRNA degradation, mRNA storage, mRNA surveillance, and RNA-based gene silencing mechanisms [23,24].

MiRNA biosynthesis is regulated at different levels. Regulation at promoter level is similar to mRNAs. The majority of DNA-binding elements and transcription factor binding sites in miRNAs promoters overlap with those that control protein-coding genes. On the other hand, transcription of primary miRNA transcripts can be dynamically regulated in response to growth factor stimulation, including platelet-derived growth factor (PDGF), transforming growth factor beta (TGF-β), VEGF or Wnt signaling [25,26,27].

Epigenetic control plays an important role in miRNA expression, which is associated to DNA methylation and histone modifications. For instance, levels of miRNA-127 are reduced due to promoter hypermethylation in bladder cancer [28], or the regulation of miRNA-1 by inhibition of histone deacetylase (HDAC) enzyme in breast and colon cancers [29,30]. Hence, regulation of miRNA expression is induced by external stimulus and can be epigenetically controlled; therefore, the condition of chromatin and cellular microenvironment can affect the expression levels of miRNA global profile.

## 3. MiRNAs Associated to Development and Progression in Cervical Cancer

It has been established that persistent HPV infection is the initial step leading to CC, but other factors are required for the development and progression of malignant phenotypes. CC is a complex disease caused by the interaction of viral, host, and environmental factors, which influence disease progression from early cervical abnormalities, to invasive and metastatic cancer. Molecular evidence emerged in the last three decades, has centered in the discussion of alterations of protein-coding oncogenes or tumor suppressor genes as the main drivers to malignant phenotype. However, the general idea of complexity in tumor cells has been significantly enhanced with the discovery of hundreds of genes producing non-coding RNAs (ncRNA). In this scenario, miRNAs (miRNAs) are considered important players regulating cellular processes such as apoptosis, cell cycle progression, metastasis and radioresistance. For this reason, miRNAs have been studied also encompassing the etiology, progression and prognosis of cancer [31].

The first evidence showing changes in the expression level of miRNAs on tumor cells was obtained from B-cell chronic lymphocytic leukemia [32]; since then, a huge amount of data produced by expression profiles of miRNAs in tumor tissues and derived cell lines, has been detected in different types of cancer. The seminal work of Lu and colleagues in 2005 showed that miRNA tumor profiles are informative, reflecting the developmental lineage and differentiation state of the tumor. Moreover, they could successfully classify each tumor type by means of miRNA profiling, whereas messenger profiles were inaccurate to do it [33].

Since then, several works have been published. In CC research, Lee and co-workers analyzed the differential expression, by means of TaqMan real-time quantitative PCR array, of 157 human mature miRNAs [34]. The investigation comprised ten tumor biopsies, staged as primary invasive squamous cell carcinomas (ISCC), and ten normal tissues. They found that 70 miRNAs were significantly differentially expressed; 68 of them were up-regulated and two were down-regulated. Among them, 10 miRNAs were over-expressed with fold changes of more than 100. The top-ten over-expressed miRNAs were miR-199-s, miR-9, miR199a*, miR-199a, miR-199b, miR-145, miR-133a, miR-133b, miR-214 and miR-127. On the other hand, the only two underexpressed miRNAs were miR-149 and miR-203. Interestingly, expression of miR-127 was significantly associated to lymph node metastasis. Finally, they chose miR-199a to block its expression by means of anti-miR-199a transfection on CC-derived cell lines, showing an important reduction on cell growth [34].

In a larger study with a sample of 102 CC tumor biopsies, the expression profile of 96 cancer-related miRNAs was analyzed by quantitative RT-PCR [35]. The researchers used a support vector machine mathematic algorithm, in order to select 10 miRNAs that could be used to predict the overall survival in the patient cohort. Among the selected miRNAs, miR-200a and miR-9 were significantly associated to overall survival. Finally, they individually transfected miR-200a and miR-9 into HeLa cells, and the expression profile of transfected cells was analyzed. By means of gene set enrichment and gene ontology based analyses, they showed that miR-200a could regulate the metastatic potential of cancer cells to migrate to distant sites; hence, miR-200a could control cancer phenotype by regulating metastasis processes, whereas genes regulated by miR-9 were involved in metabolic processes, explaining the maintenance of high metabolic rate by tumor cells, an important issue for the rapid proliferation of cervical cancer cells.

In the same research line, Pereira *et al.*, analyzed the differential expression associated with CC progression, in this case, they employed normal tissues, moderate/severe dysplasia and invasive squamous cell carcinoma infected with HPV16 sequences. Hence, they identified the altered expression of 10 miRNAs between cancer and dysplasia tissues compared to the normal cervical tissues (miR-16, miR-21, miR-106b, miR-135b, miR-141, miR-223, miR-301b, and miR-449a were significantly over-expressed). In particular, miR-21, miR-135b, miR-223, miR-301b, and miR-135b were significantly over-expressed in CC tissue compared to normal and dysplasia tissue, suggesting that the above list of miRNAs may be useful in distinguishing cervical cancer and dysplasia from normal cervical tissue [36].

Recently, the analyses by quantitative real-time reverse transcriptase PCR (qRT-PCR) of the expression of miR-100 in 125 cervical tissues including normal cervical epithelium, cervical intraepithelial neoplasia (CIN), and cervical cancer, as well as in five cervical cell lines were published [37]. MiR-100 expression exhibited a significantly and gradually reduced tendency from low-grade CIN, high-grade CIN to cervical cancer tissues, and a significant decrease in HPV positive cervical cancer cell lines. Remarkably, the expression of miR-100 negatively influenced cell proliferation, cell cycle and increased apoptosis; probably by means of PLK1 protein regulation (Polo-like kinase1). Also, it was found that PLK1 expression was negatively correlated with miR-100 expression in CIN3 and cervical cancer tissues. Finally, the authors concluded that miR-100 participates in the development of cervical cancer at least partly through loss of inhibition to target gene PLK1, which probably occurs in a relative late phase of carcinogenesis [37].

Regarding CC progression associated to HPV 16-infected samples in 18 cervical tissues, Li *et al.*, employed 18 tissues obtained from normal, CIN 2-3 and squamous cell carcinoma biopsies; using miRNAs microarray, they covered 875 human miRNAs. Interestingly, the findings showed 31 unique miRNAs with a significantly increased expression from normal to CC (17 up-regulated and 14 down-modulated). Among these, the miR-218 was the most significantly down-regulated from normal to CC, while miR-29 showed an over-expression. Furthermore, they showed an important negative correlation of YY1 and CDK6 expression with miR-29, and was suggested that the expression level of miR-29 could be regulated by HR-HPV E6/E7 [38]. Table 1 summarizes miRNA profile deregulation in premalignant and tumor CC tissues.

## 4. MiRNA Profile Regulated by HR-HPV Oncoproteins

As it has been described previously, along the CC carcinogenesis process, persistent HPV infection is a necessary etiological agent which induces cellular alterations by means of viral onco-proteins such as E5, E6 and E7, that control several important molecular routes considered as hallmarks of cancer. Although this review does not focus on HPV oncoprotein activities, we briefly summarize some of their most important biological interactions. E5 interacts with different cellular proteins contributing to the biological activity of the protein in cell transformation and evasion of the immune response. Thus the first transmembrane domain of 16E5 and HPV-31E5 interacts directly with the heavy chain component of the MHC I (Major Histocompatibility Complex class I) promoting the immunosurveillance evasion of infected cell, that could increase the extent of HPV infection [39].

**Table 1:** MiRNAs involved in the development and progression of cervical cancer.

MiRNA	Up/Down-Regulated	Cellular Process	Target Gene	Clinical Background	Refs.
miR-34a	Down-regulated	p53-dependent pathway (cell cycle progression, cellular senescence and apoptosis)	p18Ink4c, CDK4, CDK6, Cyclin E2, E2F1, E2F3, E2F5, BCL2, BIRC3, and DcR3	↓CIN I, ↓↓CIN II, ↓↓↓CIN III	[40,41]
miR-218	Down-regulated	Focal adhesion	LAMB3	↓CIN III, ↓↓↓CaCu	[42]
miR-200a, miR-205	Basal expression	Metastasis (inhibit the epithelial to mesenchymal transition)	ZEB1, ZEB2 and SIP1	CaCu → CaCu metastasis	[43]
miR-9	Up-regulated	Tumor cell metabolism (ATPase activity, Group transfer coenzyme metabolic process, Glutamine family amino acid metabolic process	No identified	Cervical cancers	[35]
miR-127	Up-regulated	Metastasis	No identified	↓NSE ‒↑↑ISCCs	[34]
miR-199a	Up-regulated	Cell growth	No identified	↓NSE ‒ ↑↑ISCCs	[34]
miR-372	Down-regulated	Cell growth (induced arrest in the S/G2 phases of cell cycle)	CDK2, Cyclin A1	Cervical normal tissue → cervical cancer tissues	[44]
miR-203	Up-regulated	Keratinocyte differentiation/maintain HPV episomes	p63-family	Normal epithelia → HPV-infected epithelia	[45]
miR-26a	Down-regulated	Cellular Growth and Proliferation	No identified	↑Normal, ↓CIN, ↓CIN III, ↓Carcinoma	[36]
miR-143	Down-regulated	Cellular Growth and Proliferation	PPAR Signaling	↑Normal, ↓↓CIN, ↓↓CIN III, ↓↓Carcinoma	[36]
miR-145	Down-regulated	Cellular Movement	IGF-1	↑Normal, ↓CIN, ↓CIN III, ↓Carcinoma	[36]
miR-99a, miR-203, miR-513, miR-29a	Down-regulated	Cell Death, Tissue Development	IGF-1, BCL2L2, VEGFA and CDK6	↑Normal, ↓CIN, ↓CIN III, ↓Carcinoma	[36]
miR-522*	Up-regulated	Cell Cycle: G2/M DNA Damage Checkpoint Regulation	No identified	↑Normal, ↑↑CIN, ↑↑CIN, ↓Carcinoma	[36]
miR-148a	Up-regulated	Tumor supresor genes	PTEN, P53INP1 and TP53INP2	↑Normal, ↑↑CIN, ↑↑CIN, ↑↑↑Carcinoma	[36]
miR-10a, miR-196a, miR-132	Up-regulated	Cell transformation and progression	(HOX) genes	↑Normal, ↑↑↑CIN, ↑↑↑CIN, ↑↑↑Carcinoma	[36]
miR-886-5p	Up-regulated	Apoptosis	BAX	↑ANTT, ↑↑↑CSCC	[46]
miR-100	Down-regulated	Growth, cell cycle, and apoptosis	PLK1	↑ Normal, ↓CIN, ↓↓Carcinoma	[37]

E5 shows a preferred membrane localization which explains some of their biological functions, for example, HPV16-E5 enhance ligand-dependent activation of the EGF-R, stimulating EGF-dependent proliferation of cultured cells [47,48,49]. E5 also can prompt apoptosis evasion by impairing tumor necrosis factor ligand (FasL), and tumor necrosis factor related apoptosis-inducing ligand (TRAIL) in cultured cells; mainly by inducing downregulation of Fas receptor and disrupting the formation of Death-Inducing Signalling Complex (DISC) activated by TRAIL [50]. In a previous study published by Greco and colleagues, they show that HPV16-positive keratinocytes expressing E5 are able to downregulate miR-203 and miR-324-5, and upregulate miR-146a [51].

On the other hand, it is known that the active expression of E6 and E7 is required to increase the proliferation capacity of malignant cells and uncoupling differentiation, through targeting prominent regulators of cell cycle control progression. The central core of the classic E6/E7 model is the binding and inactivation of tumour suppressor proteins p53 and pRb, respectively, affecting molecular pathways involved in cervical carcinogenesis. For instance, auto-proliferative signaling, growth suppressor evasion, programmed cell death evasion, and replicative immortality, among others. Moreover, it has been demonstrated that complex interactions between HR-HPV E6 and E7 involve the activation of transcription factors, such as E2F and c-Myc, which can promote the transactivation of miRNA expression [52]. Hence, it is conceivable that expression of viral oncoproteins can modulate the expression levels of miRNAs enhancing the progressive alterations leading to invasive cancer and radioresistant phenotype. In this regard, Yeung *et al.* demonstrated that miR-23b is down-regulated by E6-targeting urokinase-type plasminogen activator (uPA), which is over-expressed in CC. Therefore, decreased levels of miR-23b, enhance the expression of uPA, and thus, induce the migration of CC derived cells. Moreover, a consensus p53 binding site was detected in the promoter region of miR-23b; hence, miR-23b/uPA are involved in HPV-16 E6-associated cervical cancer development [53]. Concordantly, in a recent report Wang *et al.*, it was clearly demonstrated that radioresistant pancreatic cancer cells show reduced levels of miR-23b and increased autophagy compared to cells that are not radioresistant. Moreover, overexpression of miR-23b inhibits radiation-induced autophagy, whereas an inhibitor of miR-23b promoted autophagy in pancreatic cancer cells. Hence, overexpression of miR-23b sensitized pancreatic cancer cells to radiation. One proposed mechanism was the relationship between miR-23b and ATG12, which is overexpressed in radioresistant cells (levels of ATG12 protein are correlated with the occurrence of autophagy). Expression of miR-23b blocked radiation-induced autophagy and sensitized pancreatic cancer cells to radiation [54]. Those results corroborate the proposed hypothesis, HR-HPV oncoproteins can regulate a network of mechanisms associated to radio-resistance conducted by miRNAs. Table 2 lists the major miRNAs affected by HPV associated genes that could regulate the radioresistant phenotype observed in tumor cells.

**Table 2:** HPV-Oncoproteins are able to regulate the expression of miRNAs.

Protein	MiRNAs	Up-/Down-Regulated	Target Gen	Cellular Process	Refs.
E5	mir-146a	Up-regulated	ZNF813	Cell adhesión and cell cycle	[51]
E5	mir-324-5p	Down-regulated	CDH2, CTNNB1	Transendothelial migration	[51]
E5	mir-203	Down-regulated	p63	Cell juntion, cell migration, and cell motility	[51]
E6	mir-34a	Down-regulated	p18Ink4c, CDK4, CDK6, Cyclin E2	Cell cycle progression, cellular	[40] [41]
E6	mir-218	Down-regulated	LAMB3	No identified	[42]
E6	mir-23b	Down-regulated	uPA	Cell migration	[53]
E6/E7	mir-29	Down-regulated	YY1 and CDK6	Restrains cell cycle progression and induces apoptosis	[38]
E7	mir-15b	Down-regulated	CCNA2, CCNB1, CCNB2 MSH6 and MCM7	Recognition of mismatched nucleotides, prior to their repair, and initiation of eukaryotic genome replication.	[55]
E7	miR-15a/miR-16-1 and miR-203	Down-regulated	c-Myc, c-Myb, PPAR	Control cell proliferation, survival, and invasion	[52]

MiR-34a has been identified as a direct transcriptional target of cellular transcription factor p53 [56,57]. Transactivation of miR-34a expression is elicited by the binding of p53 to a consensus binding site present in the miR-34a promoter region. Consequently, as HPV E6 oncoprotein destabilizes p53 during virus infection, it is feasible to assume a down-regulation of miR-34a expression in most cervical cancer tissues with oncogenic HPV infection. Thus, Chang and colleagues showed that miR-34a is down-regulated in productive, pre-malignant HPV infections, cervical cancer tissues, and cervical cancer cells. MiR-34a targets multiple cell cycle components, including CDK4, cyclin E2, E2F-1, hepatocyte growth factor receptor MET, and Bcl-2 [57,58,59,60]. Therefore, it is reasonable to postulate miR-34a as a tumor-suppressor miRNA, which expression is negatively modulated by HR-HPV E6 oncoprotein. Kang and colleagues, demonstrated that mir-34a participates on the radiation-induced Notch-1 signaling pathway. They used flavonoid compounds that regulate Notch-1 as radiosensitizers in non-small cell lung cancer (NSCLC) cells. Hence, flavonoids increased the expression of tumor-suppressive miRNA, miR-34a, in a p53-dependent manner, leading to inhibition of Notch-1 expression. Consequently, reduced Notch-1 expression promoted apoptosis through significant down-regulation of the nuclear factor-κB pathway, resulting in a radiosensitizing effect on NSCLC cells [61]. As seen, HPV could either positively or negatively regulate miRNAs involved in radiation response of cancer cells through mainly inactivation of p53. The direct regulation exerted by means of viral oncoproteins is an exciting new discovery area of biomedical research.

A key process during CC progression is the proliferative capacity of differentiating epithelial cells. Regarding this, it has been observed that miR-203 is a critical molecule that regulates the transition of keratinocytes from a proliferative state in undifferentiated basal cells, to a nonproliferative status in differentiated suprabasal cells. Also, it has been suggested to promote epithelial maturation by suppressing the “stemness” potential [62]. Hence, reducing the expression of miR-203 appears to be important in facilitating the productive phase of the HPV life cycle in differentiating epithelia. One of the identified targets of miR-203 is p63, which is a transcription factor that regulates the balance between epithelial proliferation and differentiation [45]. Melar-New and Laimins recently demonstrated that E7 protein has the ability to downregulate miR-203 expression upon differentiation, which may occur through the mitogen-activated protein (MAP) kinase/protein kinase C (PKC) pathway [45]. In different studies, it has been shown that miR-203 is a potent inhibitor of stemness state; therefore, its downregulation by E7 could maintain a stem phenotype in tumor cells [62,63,64]. It has been widely demonstrated, that tumor stem cells are able to resist radio and chemotherapy. The mechanisms that support tumor radioresistance are complex and, in part, sustained by a special subpopulation of tumorigenic cell. The so-called tumor stem cells (TSC) have the exclusive ability to self-renew and repopulate the tumor, and have been reported to be less sensitive to radiation-induced damage through preferential activation of DNA damage checkpoint responses, and increased capacity for DNA damage repair. During each fraction of radiation, non-stem cancer cells die and TSCs become enriched and potentially increase in number, which may lead to accelerated repopulation [65,66,67,68]

## 5. Radio-Resistance in Cervical Cancer Could Be Controlled by MiRNAs

Radiation resistance is a current problem in the treatment and clinical management for various cancers including head and neck, pancreas, esophagus, breast, lung, and cervix, among others. For those tumors radiotherapy is the first choice treatment [69,70,71,72]. Radiation causes lethal effects on cells through the modification of expression levels of antioxidant enzymes and by generating free radicals which are a source for reactive oxygen species (ROS) and reactive nitrogen species (RNS). ROS and RNS have a direct impact damaging integrity of genomic DNA. Some proteins involved in DNA repair have been identified, as for example ATM, MRE11, RAD50, BRCA, Rad3 and DNA-dependent protein kinase. Those factors can lead to an increase in DNA repair ability, causing resistance to radiotherapy. Radioresistance of tumor cells is also associated with the expression of p53, RAS, RAF-1, BCL-2 and survivin [73,74,75]. Despite of some molecules associated with radioresistance being well known, the molecular mechanisms regulating the phenomenon remain unclear.

**Figure 3:**
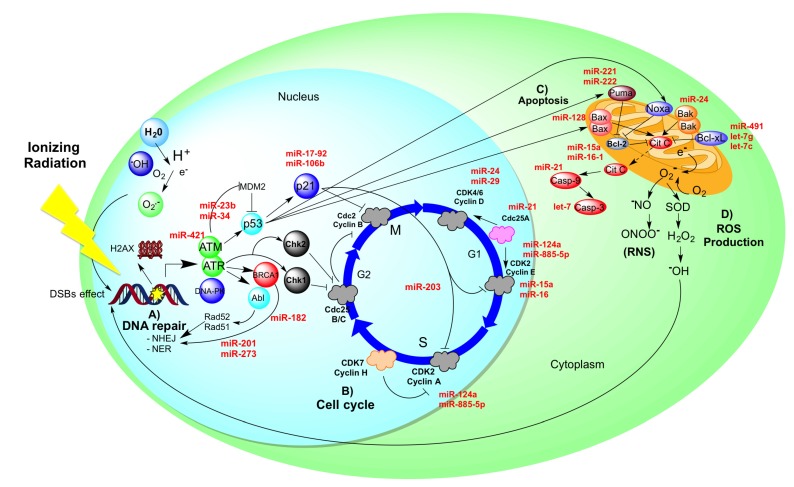
Major molecular process regulated in radioresistant tumor cells.

Using omics-methodologies to have a more robust view of the molecular process, knowledge in this field has been notably enhanced. In this context, in a seminal work comparing radiosensitive and radioresistant cervical tumors, it was identified a subset of 121 genes whose expression was significantly higher in the radiosensitive samples; whereas expression of 50 genes was down-regulated in radioresistant samples [76]. Gene-ontologies associated to this work and confirmed by others were apoptotic pathway, DNA repair mechanisms and DNA damage response, cell cycle, actin-associated and cytoskeletal proteins, and Ras-protein family [77,78]. Those results emphasize common mechanisms regulating a complex process such as radio-resistance in cervical cancer (Figure 3).

However, although such discoveries have brought partial understanding of the molecular mechanisms responsible for cellular radiosensitivity, the complete process remains to be clarified. The missing piece in this complex puzzle could be to decipher the role of specific miRNAs which expression is affected in radioresistant cells. To this respect, several reports have associated differential expression of miRNAs in radioresistant tumors and cell lines (such as lung, cervix, squamous cell carcinoma of head and neck, glioblastoma, and colon) [79,80,81,82]. Some miRNAs ave been identified as promoter of radioresistance such as miR-421 which regulates the activity of ATM protein [83], the main sensor associated to ionizing radiation response in conjunction with the ATR and BRCA. Additionally, miR-23b and miR-34a regulate the activity of the p53 protein [56,84], which is the major cellular sensor detecting damaged DNA after radiation exposure. MiR-17-92 and miR-106b affect another important protein, p21; which directly regulates the cell cycle, preventing replication via Cdc2 and cyclin B [85,86].

A significant study was done by Hu *et al.*, in which they used 102 samples of tumor from cervical cancer patients under standard treatment (radiotherapy and chemotherapy), by using miRNA expression profiling of 96 miRNAs with real-time reverse transcription-PCR. They identified five miRNAs (miR-9, miR-21, miR-200a, miR-218 and miR-203) significantly associated with radiosensitivity in cervical cancer. Using recursive feature elimination (RFE) and support vector machine (SVM) techniques, the authors identified two miRNAs with the most predictive score to radiosensitive patients, miR-200a and miR-9, which were subsequently validated by functional assays [35]. Finally, they proposed a miRNA-prediction model to identify CC patients who could fail to conventional treatment based on chemo- and radiotherapy.

Those and other similar studies provide an overview of miRNA expression profiles and the interactions between miRNA and their target mRNAs, which could expand our understanding about the roles played by miRNAs in the radioresistance process. Nevertheless, the field is still undercovered, as the majority of studies are descriptive and the catalogue of miRNAs associated to radioresistance could be increased notably; validation by means of functional studies is a mandatory task in order to have a complete picture of the biological role of selected miRNAs. An integral understanding of the regulation networks controlled by miRNA-target genes which are involved in the process of radioresistance, represents a major challenge for many research groups. The use of new throughput technologies seems to be possible and the use of integrative genomics strategies are necessary with a focus on establish and understand networks regulated by miRNAs.

## 6. Summary and Prospects

Cervical cancer remains as a leading cause of morbidity and mortality for women worldwide. Current conventional treatment includes radiotherapy, however a substantial percentage of patients have unresponsive tumors presenting mechanisms that allow them to escape from the effects of radiation. There is a need for robust biomarkers to optimize therapy and to use more aggressive therapeutic agents, especially in developing countries where a large number of women are already CC patients and mortality is closely linked to late diagnosis of neoplasias and failure of treatment. Viral oncoproteins have the ability to modify the expression rate of specific miRNAs that could be associated to radio-response in resistant cells. This review has emphasized the role of miRNAs that could regulate radio-resistance mechanisms; hence, the evaluation of those miRNAs as potential biomarkers opens up new horizons for cervical cancer prognosis and therapy.

Cervical cancer is a complex disease in which progression from normal infected cells to pre-neoplasic lesions, metastatic disease, and clinical response to radiotherapy, are affected by several factors, not only associated to HR-HPV infection and E6/E7 oncoprotein interaction with cellular components. In this scenario, miRNA could be a key fact in the explanation of these complex phenotypes, such as radioresistance. Hopefully, in the near future we can bring more discoveries on miRNAs science to develop molecular markers associated to cancer progression, clinical outcome, or probably to use them not only to block E6 and E7 expression in cervical cancer cells, but also to reprogram the cancer cell.
